# Progress in Diseases of the Nervous System

**Published:** 1903-07-25

**Authors:** 


### Progress in Diseases of the Nervous System.

**Carcinoma and Trophic Areas.**—Cheatle[1] has recently pointed out that in a certain proportion of cases of carcinoma there is a marked relationship between the spread of the primary focus and the distribution of nerves and trophic areas. This incidence of cancer within a nerve area is probably not a fortuitous one, but may be due to the direct or indirect nervous influence over that area. In the case of rodent ulcer of the face, for instance, where there is an extensive lesion there is an absence of spread to those areas supplied by the cervical spinal nerves. Again, where the disease begins at the meeting place of two or more peripheral nerve-areas they tend to be mapped out before the disease spreads to others; should such areas be situated on one side at the middle line the corresponding and contiguous areas of the opposite side are frequently attacked and will then produce a bilateral lesion which appears central. The disease is apt to begin at the point where the particular nerve becomes subcutaneous, *e g.*, in the case of the infra-orbital nerve. The same thing is true of Hutchinson's crateriform ulcer and typical squamous epithelioma. In the tongue is seen remarkable limitation occurring at the middle line when cancer begins on the side of that organ. There are reasons for inferring that carcinoma may originate in a nerve area as a result of the nervous influence over that area. Thus, though rodent ulcer is usually single, yet there are many recorded in which the area of the V nerve is the seat of multiple discrete rodent ulcers, either multiple on the area of one V nerve, or multiple on the areas of the two V nerves. Carcinoma belongs essentially to the period of bodily degeneration; and such degeneration may affect a nerve-root and thus bring about changes in the corresponding area, which may have something to do with the genesis of cancer in that area.

**Herpes Brachialis with Deltoid Paralysis.**—Pearson[2] has published the case of a man who developed herpes of the left arm, extending from the shoulder to the wrist, in distribution corresponding to the nerve roots of the fifth cervical to the second dorsal nerves. The deltoid and latissimus dorsi muscles became completely paralysed and partially atrophied, whilst the other muscles of the arm were flabby and rather wasted. The herpes gradually healed, and subsequently the deltoid and latissimus began to regain power. The electrical reactions were normal. This rare association of paralysis with herpes is probably due to the fact that the lesion of herpes is vascular in origin, and in such cases affects the anterior as well as the posterior roots.

**Cytodiagnosis of Affections of the Spinal Cord.**—Numerous French observers[3] have observed, especially in case of tabes dorsalis, cell-elements in the cerebro-spinal fluid withdrawn by lumbar puncture. In nearly all cases of that disease numerous lymphocytes are to be found, whereas in the normal cerebro-spinal fluid they are either absent or very rare. They are also abundantly present in meningeal tuberculosis, syphilitic meningo-myelitis, and in general paralysis of the insane. Their presence is an indication of a simple process of irritation. The presence of polymorphonuclear leucocytes indicates a state of congestion or inflammation, as these elements come only from the blood vessels by diapedesis. On the other hand in hysteria, epilepsy, neurasthenia, and typical polyneuritis there is no such lymphocytosis.

**Reaction of the Blood in Nervous Diseases.**—Pugh[4] has examined the alkalinity of the blood in 40 cases of epilepsy with interesting results. It was found that the typical condition was a low average alkalinity in the period between epileptic fits; secondly, a sudden and pronounced fall immediately prior to the fit; and thirdly, a further diminution soon after the fit was over. The first condition may be explained by the gradual accumulation of toxins of an acid nature in the blood, probably arising from deficient metabolism of the body tissues generally. The third condition was regarded as due to the production of sarcolactic and carbonic acids during the violent tonic and clonic convulsions of the fit. The second condition is very difficult to account for, though it certainly suggests the presence of some poison of an acid nature in the blood as the immediate precursor of the fit.

**Progressive Muscular Atrophy.**—Campbell[5] has made an important addition to our knowledge of the pathology of this affection by showing that the cortex cerebri is affected. In the two cases examined, the muscles, the peripheral nerves, the spinal cord and the bulb showed typical alterations; whilst in that part of the cortex which is the normal position of the giant cells of Betz—*i.e.*, the ascending frontal gyrus and the paracentral lobule, scarcely any of these elements could be discovered and their disappearance was associated with a general distortion of the cells which remained, atrophy of efferent fibres and distension of cortical blood vessels. The ascending parietal and other neighbouring convolutions were relatively healthy. The view that the spinal changes in progressive muscular atrophy were primary was rendered unstable by these findings, for a retrograde destruction of the cells of Betz could not take place during the progress of a disease which did not last longer than three years. The alternative views are either that the cortical changes are primary or else in progressive muscular atrophy there is a general and more or less simultaneous affection of the whole system of motor neurons. The retention of the muscular sense in the disease, in view of these results, might be used as an argument against the theory of the mixed sensori motor function of the central convolutions.

[1] Brit. Med. Jour., April 18. [2] Lancet, May 2. [3] Lancet, May 30. [4] Jour. of Mental Science, January. [5] Lancet, April 4.

